# Spread of Cystic Echinococcosis in Pakistan Due to Stray Dogs and Livestock Slaughtering Habits: Research Priorities and Public Health Importance

**DOI:** 10.3389/fpubh.2019.00412

**Published:** 2020-01-29

**Authors:** Aisha Khan, Haroon Ahmed, Sami Simsek, Muhammad Sohail Afzal, Jianping Cao

**Affiliations:** ^1^Department of Biosciences, COMSATS University Islamabad, Islamabad, Pakistan; ^2^Key Laboratory of Parasite and Vector Biology, MOH, Shanghai, China; ^3^Department of Parasitology, Faculty of Veterinary Medicine, University of Firat, Elazig, Turkey; ^4^Department of Lifesciences, University of Management & Technology, Lahore, Pakistan; ^5^WHO Collaborating Centre for Tropical Diseases, Shanghai, China; ^6^Chinese Center for Disease Control and Prevention, National Institute of Parasitic Diseases, Shanghai, China

**Keywords:** cystic echinococcosis, *Echinococcus granulosus*, livestock, dog, public health, Pakistan

## Abstract

**Background:** Cystic echinococcosis (CE) is a global zoonotic parasitic disease caused by the larval stage of *Echinococcus granulosus* and it has been reported from both livestock and humans in Pakistan. The definitive host of *E. granulosus* is the dog, and the large number of stray dogs in Pakistan contributes to the spread of CE. However, there is little information between stray dogs and CE relation in the country.

**Methods:** During the study, total 123 butcher's shops and abattoirs were included for collection of data relating to the hydatid cyst prevalence in slaughtered animals (sheep, goat, cattle, and buffaloes). The number of animals slaughtered in each butcher's shop during sampling period was also recorded, and the association of the shop environment with dogs was inspected.

**Results:** Data was collected for CE from 123 butcher's shops in Rawalpindi and Islamabad, Pakistan. The slaughtering rate the in the butcher's shops was 2–10 animals/day including sheep/goat/cattle and buffaloes. The overall prevalence of CE in all examined animals was 2.77%. In buffaloes the higher prevalence was recorded as compared to other hosts. The findings showed that lung and liver were most affected organs and majority (59%) of the cysts were fertile in infected animals. The presence of a large number of stray dogs were an important factor in the spread of CE. They were rarely vaccinated, have easy access to infected offal at slaughtering site and had insufficient or inappropriate anthelmintic treatment.

**Conclusions:** The most pressing need is to raise public awareness of this huge problem by considering CE a major ailment and promoting the collection and mapping of epidemiological data. Efficient CE control is required, especially treating dogs with antiparasitic drugs, for which government support and affiliation with the veterinary sector is essential.

## Introduction

Echinococcosis is one of the 20 neglected zoonotic diseases (NZD) prioritized by the World Health Organization (WHO) (1). Cystic echinococcosis (CE) is a globally NZD caused by the dog tapeworm *Echinococcus granulosus*. The global annual infection rate is 1.2 million, the annual death rate is about 2.2%, and an estimated 3.6 million disability-adjusted life years (DALYs) are lost because of this disease per annum (2). In addition, CE is responsible for over US$ 3 billion expenses every year (3). CE is more prevalent in areas where people survive on animal husbandry and agricultural activities (4), and the rate is higher in nomadic and semi-nomadic populations due to this lifestyle (5).

Conditions such as poor hygiene and failure to wash contaminated food facilitate the spread of CE infection in the human population (6). CE transmission from food to humans is common in areas where people usually consume raw vegetables; most are cultivated in open fields where stray dogs roam freely and contaminate the vegetables by dropping feces containing *E. granulosus* eggs (7). One of the major risk factors for CE infection is open slaughtering of livestock without veterinary supervision. Due to lack of supervision, infected offal is ingested by dogs, which, as the intermediate host, spread infected eggs to the environment. The study aimed to analyze CE prevalence, presence of stray dogs and their association with slaughtering habits in abattoirs /butcher shops in the study area.

## Materials and Methods

### Study Area

A study was conducted in Islamabad and Rawalpindi districts of Pakistan.

### Topography

Islamabad, the capital city of Pakistan, is located in Pothohar Plateau (33.43°N 73.04°E) at 540 m (1,770 ft.) above the sea level. 505 km^2^ of this area is urban whereas 401 km^2^ of is rural (8). Adjoining Islamabad is the city of Rawalpindi and both the cities are often referred to as the twin cities, 84% of the population here is Punjabi, 9% Pashto and 7% others. Rawalpindi is located at an elevation of 508 m and spans over an area of 259 km^2^ (9).

### Study Duration

The data was collected from January to July, 2017(for 6 months). Butcher shops in different areas of Rawalpindi and Islamabad were visited twice per month to collect the data on prevalence and presence of stray dogs in the slaughterhouses.

### Study Design

A cross-sectional survey was designed to get the recent data hydatid cyst incidence. The data was collected from butcher shops of the twin cities. Questionnaire was designed for butcher shops/slaughterhouses among urban and rural areas which was descriptive in nature. The information about stray dogs present along the territory of slaughterhouses/butcher shops were recorded.

### Data Collection Methods

The data on presence of stray dogs, CE prevalence in animals, as well as on socio-demographic characteristics was collected using questionnaires. Moreover, data was analyzed to determine the factors associated with the risk of CE. As there is no local specific name of this disease, pictures of cysts in animal organs and of infected humans were shown to the participants to identify the disease better. The knowledge of the participants was measured as binary outcomes (10, 11).

### Laboratory Investigations

In order to examine hydatid cysts properly, following parameters were carried out: Types of cysts (sterile, fertile, calcified, or under-developed), organ specificity (lungs and liver), and prevalence of hydatidosis. Presence of cysts in different organs was analyzed by routine post-mortem of the carcass. The cysts were dissected and collected into sterile containers separately on organ basis for further description.

### Cyst Characterization

Sterile scalpel blades were used for cyst incision. The fluid present inside these cysts was used to check the existence of protoscoleces either in the form of brood capsule (closes to the germinal layer) or in the cyst fluid considering as a fertility indicative. Viability test was performed on fertile cysts. In viability test a drop of fluid from cyst containing the protoscoleces was observed under microscope to check amoeboid like peristaltic movements. For clear microscopic observations equal volume of 0.1% aqueous eosin solution was also mixed with equal volume of fluid containing the protoscoleces. Sterile hydatid cysts were characterized on the basis of inner lining, generally smooth with a slight turbid enclosed fluid otherwise rough calcified cyst with no or less fluid (12). Calcified cysts were coarse and nodular having an internal chamber with calcified or chalky deposits in the cyst wall. Underdeveloped cysts were small 1–2 mm in size, defined germinal layer are firm in texture with very little fluid but presence of protoscoleces was not observed (13).

### Morphology of Protoscoleces

Polyvinyl-lactophenol was used for mounting protoscoleces cysts. Hooks damage was prevented by applying gentle pressure on cover slip. A calibrated eye-piece micrometer was used for all measurements under oil immersion. Morphometric analysis was done as described by Hobbs et al. (14).

### Data Analysis

Data was analyzed as described previously (15).

## Results

Data was collected for CE from 123 butcher's shops in Rawalpindi and Islamabad, Pakistan. The slaughtering rate in the butcher's shops was 2–10 animals/day including cattle, goat, sheep, and buffaloes. Overall prevalence of CE in the slaughterhouses/butcher shops was 2.77% (300/10,800) according to this survey. Prevalence was higher in buffaloes followed by cattle, sheep, and goat, respectively. The site of infection, number of cysts and kind of cysts are shown in Table 1.

**Table 1:** Overall prevalence (%) of hydatidosis in various organs of slaughtered Cattle, Buffalo, Goat, and Sheep.

**Host**	**Overall prevalence**			**Site of infection**			**No. of cysts (%)**		**Kind of cysts (%)**			
	***N***	**Infected**	**Frequency (%)**	**Lung**	**Liver**	**Others**	**Single**	**Multiple**	**Fertile**	**Sterile**	**Calcified**	**Undeveloped**
Cattle	3,845	132	3.43	✓	✓	✓	103 (78)	29(22)	73 (55.3)	31 (23.48)	19 (14.39)	9 (6.81)
Buffalo	1,103	58	5.25	✓	✓	✓	47 (81)	11(19)	48(82)	5(8.62)	4(6.89)	1(1.72)
Goat	4,307	76	1.76	✓	✓		68 (89)	08 (11)	37 (48.68)	21 (27.63)	15 (19.73)	3 (3.94)
Sheep	1,545	34	2.20	✓	✓		34 (100)	–	19 (55.88)	3(8.82)	7(20.58)	5(14.7)
Total	10,800	300	2.77				252 (84)	48 (16)	177 (59)	60 (20)	45(15)	18(6)

### Rostellar Hook Morphology

The parameters which were observed to check protoscolex rostellar hook morphology in infected animals were total hooks number, their total length (μm) of hooks and blade length (μm) as shown in Table 2.

**Table 2:** Rostellar hooks morphology of protoscoleces in infected animals.

**Parameters**	**Mean + S.E**			
	**Cattle**	**Buffalo**	**Goat**	**Sheep**
Total No. of Hooks (NH)	29.21 ± 1.13	26.03 ± 1.17	21.00 ± 1.06	27.80 ± 1.11
Large Hook Length (LTL) (μm)	24.02 ± 1.03	18.37 ± 0.96	27.12 ± 0.91	19.78 ± 1.02
Large Hook blade Length (LBL) (μm)	15.21 ± 0.44	16.02 ± 0.54	9.77 ± 0.57	10.06 ± 0.38
Small Hook Length (STL) (μm)	19.54 ± 1.03	17.97 ± 1.00	11.22 ± 0.77	13.15 ± 0.72
Small Hook Blade Length (SBL) (μm)	8.9 ± 0.56	6.9 ± 0.30	9.30 ± 0.38	7.2 ± 0.37

### Total Number of Hooks (NH)

Protoscoleces hooks number was observed and it was found that total number was 29.21 ± 1.13 in cattle origin, 26.03 ± 1.17 in buffalo origin, 21.0 ± 1.06 in goat origin, and 27.80 ± 1.11 in sheep origin as shown in Table 1. The study results indicated that the maximum number of hooks were observed on protoscoleces of sheep origin and minimum on those of goat origin.

### Large Hook Total Length (LTL) (μm)

Protoscoleces large hooks was observed for total length (micrometers, μm) and it was found that it was 24.02 ± 1.03 in cattle origin, 18.37 ± 0.96 in buffalo origin, 27.12 ± 0.91 in goat origin, and 19.78 ± 1.02 in sheep origin. In goat origin large hook length was maximum (27.12 ± 0.91) and in case of buffalo origin it was minimum (18.37 ± 0.96).

### Large Hook Blade Length (LBL)(μm)

Protoscoleces blade length of large hooks on was observed as 15.21 ± 0.44 in cattle originated infections, 16.02 ± 0.54 in buffalo originated infections, 9.77 ± 0.57 in goat origin, and 10.06 ± 0.38 in sheep origin as shown in Table 1. It is clear from these values that buffalo originated infection LBL was found maximum (16.02 ± 0.54) and in goat originated it was minimum (9.77 ± 0.57).

### Small Hook Total Length (STL) (μm)

Protoscoleces of small hooks total length was observed as 19.54 ± 1.03 in cattle, 17.97 ± 1.00 in buffalo, 11.22 ± 0.77 in goat, and 13.15 ± 0.72 in sheep origin as shown in Table 1. In cattle originated STL was maximum (19.54 ± 1.03) and in case of goat origin it was minimum (11.22 ± 0.77).

### Small Hook Blade Length (SBL)(μm)

Protoscoleces small hooks blade length on was recorded as 8.9 ± 0.56 in cattle origin, 6.9 ± 0.30 in buffalo origin, 9.30 ± 0.38 in goat origin, and 7.2 ± 0.37 in sheep origin. In goat origin SBL was maximum (9.30 ± 0.38), while in case of buffalo origin it was minimum (6.9 ± 0.30).

**Figure 1:**
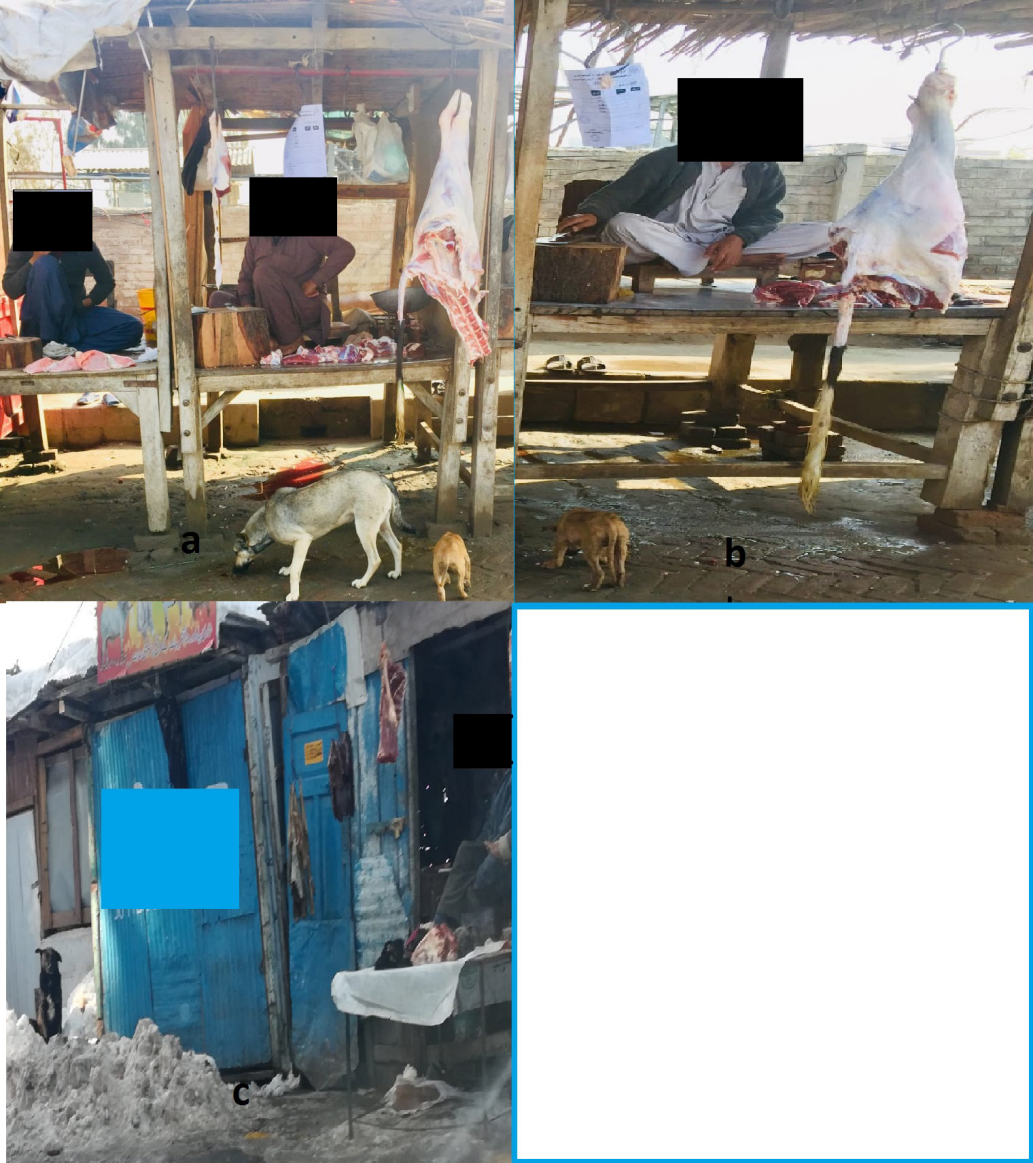
**(A-C)** Showing the association and access of stray dogs to infected offal's at butcher shops.

In the present study the number of stray dogs were recorded in all 123 slaughterhouse/butcher shops. It ranged from 1 to 5 dogs/site. The main contributing factor to the spread of CE was the large number of stray dogs (Table 3); they were rarely vaccinated, have easy access to infected offal in rural areas (Figures 1A–C), and had insufficient or inappropriate anthelmintic treatment.

**Table 3:** Potential risk factors analysis of CE.

**S. No**	**Risk Factors**	**Responses**	
		**Yes**	**No**
1	Ever heard about Zoonoses	04	119
2	Presence of stray dogs inside the slaughter house/butcher shop	107	16
3	Proper facilities to dispose animals offals in slaughter house/butcher shop	04	119
4	Discard of infected organs (Lungs/Liver) at the site of slaughtering	121	02
5	Access of stray dogs to the infected organs	121	02
6	Stray dogs were fed with useless meat (Infected)	112	11
7	Stray dogs are ever vaccinated	02	122
8	Cystic Echinococcosis is spreaded from dogs?	01	23
9	Veterinary supervision of slaughtered animals	04	119
10	Health education to butchers	0	123
11	Anthelminthic treatment of stray dogs	0	123

In addition, there were few municipal slaughterhouses, limited veterinary supervision and inspection of slaughterhouses, few facilities for the disposal of infected offal, and there was home or illegal livestock slaughtering, and lack of health education. It was observed that stray dogs have a close association with the slaughtering sites and increase the chances to get infected with CE. The finding of this study has showed that stray dogs (range 1–5) were present in the territories of all the butcher shops/slaughter houses that has an open access to infected offal of the slaughtered livestock. These stray dogs are not treated with any antiparasitic drug.

## Discussion

Cystic echinococcosis (CE) is a chronic larval cestode infection caused by *E. granulosus* in humans and domestic livestock, principally transmitted by an intermediate host (16). CE is recognized as a neglected disease of public health significance worldwide, particularly in low-income countries (17). Pakistan is a country with low socioeconomic development and the hygiene conditions are poor. Poor hygiene conditions such as no proper hand washing, no water boiling, lack of proper cleanliness of shops and surrounding areas, eating of contaminated food and raw vegetables, and feeding dogs meat infected with cysts are involved in the prevalence of CE in humans. The epidemiological studies showed that CE is highly prevalent r in third world countries (18). The higher prevalence of CE in Pakistan might be due to inappropriate waste dumping, poor social-economic condition of the country, very poor sanitary system, and unorganized slaughtering. In addition to these factors personal unhygienic situation is also playing a crucial role (19).

The findings showed that overall prevalence of CE was 2.77%. The prevalence was higher in buffaloes followed by cattle, sheep, and goat, respectively. In Pakistan the first incidence of CE in intermediate hosts was explored in 1968. The prevalence of *E. granulosus* was 35% (52/148) in buffaloes and 27% (17/62) in cattle (10).

In current study, lung and liver was most affected organ as compared to others. The lung wise prevalence was 30.9, 22.8, and 58.8%, in cattle, buffaloes, and camels, respectively while in liver it was 21.42, 17.47, and 26.4% in cattle, buffaloes, and camels, respectively (11). The prevalence of hydatid cyst in liver, lung, spleen, heart, and kidneys was 25.31, 47.31, 1.83, 0.06, and 0.51%, respectively (15). In sheep and goat, the prevalence was 8.25 and 8.05%, respectively (20). In a comprehensive survey, the overall prevalence of hydatidosis was 6.67% in livestocks (21). Mustafa et al. (22), reported that the prevalence of hydatid cysts as 3.24, 2.44, and 2.44% in sheep, goats and cattle, respectively while Tasawar et al. (23) reported the prevalence of 7.39% in sheep and 10.69% in buffaloes of Multan, Punjab, Pakistan. Previously it was shown that hydatid cyst prevalence was between 5 and 46% in livestock species (24).

A report from Lahore showed that hydatidosis is prevalent in sheep (8.85%) and in goats (6.21%). This survey was conducted to determine the organ specificity of hydatidosis, organ wise distribution of hydatidosis showed that in goats 40.56% in liver, followed by 34.38% in lungs, 16.95% in lungs and liver together, and 0.49% in spleen. In sheep, highly infected organ was lungs whereas liver was most infected organ in goats (20). Sheep and goat liver hydatid cyst prevalences were 46.74 and 23.28% and the rates in lungs were 17.37 and 13.68%, respectively (25).

Similarly, frequency of fertile cysts was higher as compared to sterile, calcified, and underdeveloped cysts, respectively. Hydatid cysts can be categorized as non-viable, viable, and fertile (26). Only the fertile cysts carry the active form of the parasite protoscoleces (27). The cysts diameter was 2–30 cm and it is as the inner layer from where larvae grow (28).

Zoonotic helminthes (*Toxocara* spp. and *Echinococcus* spp.) can transmit to humans by dogs and cats (29). Globally, human and dog interaction cause significant social, economic and public health issue mainly the zoonotic diseases (30). Dogs play crucial role in spread of many zoonotic infectious diseases (31). Higher population of stray dogs is one of the main contributing factors in spread of CE in Pakistan. They are infrequently vaccinated and easy access to infected offal. Poor hygienic conditions, lack of veterinary supervision and inspection of slaughterhouses, home or illegal livestock slaughtering occurs, and there is a lack of health education due to poverty (6).

The dog population depends on the accessibility of resources (for example, shelter, food, and water) (32). Although the actual number of stray dogs worldwide is not known, of the 500 million dogs in the world, around 75% are thought to be stray (33). Stray dogs survive consists of edible debris and contributions from human beings (34). Dog populations is directly linked with the size of the local human population (35).

Stray dogs are one of the important reservoirs for the transmission of zoonotic helminthes that are of public health concern especially *Echinococcus* specie. A study from Karachi (the biggest city of Pakistan), shows that among selected dogs presence of intestinal helminthes was confirmed 99% dogs and 7% carried *E. granulosus* (36).

To attain effective control of CE, it is essential to raise knowledge and awareness regarding hazardous practices and defensive measures against the disease within the community. Pakistan, being a developing country, is densely populated and socioeconomically poor. Overall poor sanitary system in Pakistan is very poor and majority of the inhabitants lives in crowded area. Rural inhabitants mainly survive on small-scale agriculture and farming. Laborers working in the fields often interact with animals and, due to illiteracy, have limited knowledge of health and hygiene and therefore are often infected by *Echinococcus* spp. (37).

In the early years of the twenty-first century, CE contributed a major global disease burden; it is one of the 12 commonest NZDs (38). It is very difficult to regulate the control of NZDs, particularly when curing humans does not prevent transmission; moreover, treatment of livestock is perceived as a low priority because the livestock hosts are usually asymptomatic (39). Since 1960, several intervention programs have demonstrated effective control of *E. granulosus* transmission, leading to a significant reduction of CE and improved public health (40). Despite these intervention programs, further work is still necessary. Thus, at present, we recommend increasing the awareness of the seriousness of CE and promoting the collection and mapping of epidemiological data. Efficient CE control requires government support and affiliation with the veterinary sector.

## Conclusion

In countries with a high number of stray dogs, such as Pakistan, and where the public education level is low, the first task for CE control should be to raise public awareness and try to prevent infected offal from being fed to dogs. Field studies should be conducted on this subject, training seminars should be given, information should be given to children in primary schools, butchers should be trained, the community should be informed by imams in mosques, and informative TV and radio programs should be broadcast.

## Data Availability Statement

The data used and/or analyzed during the current study are available from the corresponding author on reasonable request.

## Ethics Statement

The animal study was reviewed and approved by the Departmental Ethics Review Board (ERB) at the COMSATS University Islamabad (CUI), Pakistan, under ERB/18/72. This study was carried out in strict accordance with the recommendations of the guide for the care and use of laboratory animals.

## Author Contributions

AK and MA collected the data and wrote the paper following discussions with HA and SS. SS and JC also revised the paper and improved the technical quality of the manuscript. AK and JC contributed reagents and materials. All authors approved the final version of the paper.

### Conflict of Interest

The authors declare that the research was conducted in the absence of any commercial or financial relationships that could be construed as a potential conflict of interest.

## Abbreviations

WHO: World Health Organization
NZDs: Neglected Zoonotic Diseases
CE: Cystic Echinococcosis
DALYs: Disability Adjusted Life Years
